# CTG-Repeat Detection in Primary Human Myoblasts of Myotonic Dystrophy Type 1

**DOI:** 10.3389/fnins.2021.686735

**Published:** 2021-06-28

**Authors:** Stefan Hintze, Raphaela Mensel, Lisa Knaier, Benedikt Schoser, Peter Meinke

**Affiliations:** Department of Neurology, LMU Klinikum, Friedrich-Baur-Institute, Ludwig-Maximilians-University Munich, Munich, Germany

**Keywords:** myotonic dystrophy type 1, primary human myoblast cultures, CTG-repeats, DMPK, repeat detection

## Abstract

Myotonic dystrophy type 1 (DM1) is an autosomal dominant multisystemic disorder caused by unstable CTG-repeat expansions in the *DMPK* gene. Tissue mosaicism has been described for the length of these repeat expansions. The most obvious affected tissue is skeletal muscle, making it the first target for therapy development. To date there is no approved therapy despite some existing approaches. Thus, there is the demand to further advance therapeutic developments, which will in return require several well-characterized preclinical tools and model systems. Here we describe a modified method to identify the CTG-repeat length in primary human myoblasts isolated from DM1 patients that requires less genomic DNA and avoids radioactive labeling. Using this method, we show that primary human DM1 myoblast cultures represent a population of cells with different CTG-repeat length. Comparing DNA from the identical muscle biopsy specimen, the range of CTG-repeat length in the myoblast culture is within the same range of the muscle biopsy specimen. In conclusion, primary human DM1 myoblast cultures are a well-suited model to investigate certain aspects of the DM1 pathology. They are a useful platform to perform first-line investigations of preclinical therapies.

## Introduction

Myotonic dystrophy type 1 (DM1) is caused by CTG-repeat expansions in the 3′ UTR of the *DMPK* gene, which are inherited autosomal dominant. Clinically, DM1 is a slowly progressing multisystemic disorder characterized by myotonia, muscle weakness, cataracts, cardiac arrhythmia, and cardiomyopathy, insulin insensitivity and diabetes, testicular atrophy, hypogammaglobulinemia, and involvement of the central nervous system (Udd and Krahe, 2012; Meola and Cardani, 2015; Wenninger et al., 2018). Due to the predominant muscle involvement and the approximate prevalence of 1:8,000 (Faustino and Cooper, 2003; Wheeler, 2008), DM1 is considered to be the most frequent muscular dystrophy in adulthood. There is broad variability of the clinical manifestation of the disease, which ranges from congenital to late adult onset (Echenne and Bassez, 2013; De Antonio et al., 2016; Ho et al., 2019). This can be partly explained by the length of the inherited repeat length, which shows a strong correlation with the age of onset (Cumming et al., 2019; Overend et al., 2019), although there are other contributing factors (Brunner et al., 1993).

Up to 35 CTG triplets in blood derived DNA are normal, a repeat length between 35 and 49 is considered to be a premutation (Udd and Krahe, 2012). Between 50 and ∼150 repeats have been observed in a mild expression of the phenotype and ∼100 to ∼1,000 CTG repeats were identified in patients with classical DM1. Repeats consisting of more than 1,000 CTG–triplets result in congenital DM, the most severe expression of the disease (Redman et al., 1993). There is a somatic instability of the repeat expansion which depends on age and repeat size (Wong et al., 1995b) and results in mosaicism (Monckton et al., 1995a). In skeletal muscle the repeat size has been shown to be between 3– and 25–fold higher as in leukocytes (Thornton et al., 1994; Nakamori et al., 2013).

Clinical testing for DM1 is challenging due to the nature of the mutation. The standard method to detect DM1 repeat expansions is still Southern blot of genomic DNA, which is usually performed on DNA isolated from leukocytes (Brook et al., 1992; Kamsteeg et al., 2012). The disadvantages of this method are that it requires large amounts of DNA and that the detection is done by radioactive labeling, which warrants special safety measures. Other methods used are PCR and fragment size analysis by capillary electrophoresis, or triplet–primed PCR followed by fragment size analysis or melt curve analysis (Warner et al., 1996; Kamsteeg et al., 2012; Singh et al., 2014; Turner and Hilton–Jones, 2014; Leferink et al., 2019).

To date there is no therapy available for DM1. The most advanced approach, an antisense–oligonucleotide treatment used for post–transcriptional silencing of *DMPK*, failed in a first clinical trial to reach the sufficient concentration in muscle due to inadequate biodistribution (LoRusso et al., 2018). Thus, there is a demand for further work on development of therapies. This requires the accessibility of suited preclinical tools and model systems, which must be well–characterized. Available DM1 models include animal models like fruit flies, zebrafish, and mice (Sicot and Gomes–Pereira, 2013; Plantié et al., 2015; Souidi et al., 2018), immortalized human cells (Pantic et al., 2016; Arandel et al., 2017), and primary human cell cultures (Savkur et al., 2001; Renna et al., 2017; Hintze et al., 2018).

Considering the predominant muscle involvement in DM1, the usage of primary human myoblasts has several potential advantages. As they can be obtained from several patients, a number of those cultures can be considered to stratify phenotypic variability observed in DM1 patients. Furthermore, they differentiate into myotubes, thus shifting the gene expression profile toward the mature muscle, and proliferating cells allow to investigate cell cycle effects. The latter is also a disadvantage, primary cells enter into replicative senescence after a define number of divisions which is inversely correlated with the age of the donor (Hayflick, 1965). For muscle cells there are roughly 15–20 divisions possible starting from a single satellite cell (Renault et al., 2000), but this turnover number is reduced in DM1 due to premature senescence (Bigot et al., 2009). While this reflects on premature aging aspects of DM1 (Meinke et al., 2018) it also restricts the amount of available material.

Here we describe the characterization of the extended CTG–repeat in primary human myoblasts cultures gained from adult onset DM1 patients, using an adaption of a small–pool PCR/Southern blot protocol (Gomes–Pereira et al., 2004) to non–radioactive labeling. This allows to test the DM1 repeat length using less material without special safety precautions and hopefully extends the accessibility of well–characterized preclinical model systems for testing new therapeutic avenues.

## Methods

### Tissue Culture

Primary human myoblasts (Table 1) were obtained from the Muscle Tissue Culture Collection (MTCC) at the Friedrich–Baur–Institute (Department of Neurology, LMU Klinikum, Ludwig–Maximilians–University, Munich, Germany). All materials were obtained with written informed consent of the donor. Ethical approval for this study was obtained from the ethical review committee at the Ludwig–Maximilians–University, Munich, Germany (reference 45–14).

**TABLE 1:** Primary myoblast cultures used in this study.

	Phenotype	Age of onset	Age at biopsy	Sex	Source of the muscle biopsy	CTG–repeat length in	
						Blood	Muscle
DM1–1	DM1	3rd decade	42	♂	Biceps brachii muscle	50–70	
DM1–2	DM1	2nd decade	33	♀	Unknown	300–500	
DM1–3	DM1	2nd decade	34	♂	Deltoid muscle	240–430	∼600
Con–1	Unaffected		43	♂	Biceps brachii muscle	–	
Con–2	Unaffected		36	♀	Tibialis anterior muscle	–	

Primary human myoblast from DM1 patients were obtained from patients with adult onset (Table 1). Primary human control myoblasts were obtained from individuals who underwent standard diagnostics due to some muscular issues, but in which neuromuscular disorders have been excluded.

Myoblasts were grown at 37°C with 5% CO_2_ in 10 cm tissue culture plates. For cell growth skeletal muscle cell growth medium (PeloBiotech, Munich, Germany), supplemented with 40 U/ml Penicillin and 0.04 mg/ml Streptomycin was used, cells were kept from reaching confluency by splitting at a density of about 80%. Myoblasts were harvested between passages 6 and 8.

### DNA Isolation

To obtain DNA from the primary myoblast cultures cells were trypsinized until they detached from the plate. Cells were pelleted by centrifugation (260 rcf, 5 min) and the supernatant was discarded. The cell pellet was washed twice with PBS, each washing step followed by centrifugation (16,100 rcf for 1 min) and stored at −80°C till further processing. For DNA isolation the Quick—DNA^TM^ Miniprep Plus Kit (ZymoResearch) was used according to the manufacturer’s instructions. DNA concentration was measured by a Nanodrop (Thermo Fisher Scientific).

### Size-Marker

**FIGURE 1:**
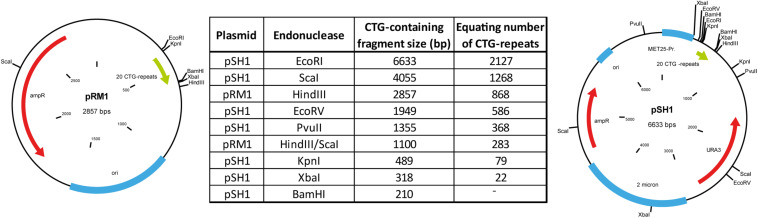
Plasmids used to create the size marker by restriction digest with endonucleases and the resulting sizes of the CTG-repeat containing fragments.

To generate a marker that allowed us to measure the number of CTG-repeats we first amplified a 171 bp DNA fragment, containing 20 CTG repeats, using the DM-C and DM-DR primer published by Monckton et al. (1995a). This amplicon was then blunt-end cloned into a pUC19 plasmid. We named the resulting plasmid pRM1. By restriction digest (EcoRI/HindIII) and ligation of the cut-out fragment (containing the CTG repeats) into a p426MET25HA backbone we created the plasmid pSH1. Both plasmids were amplified in *E. coli*, purified, and digested using different endonucleases. These specific digestions gave rise to different DNA fragments of defined size containing the 20 CTG repeats (Figure 1).

### Dig Probe

Dig-probes were designed to directly target the repeat and ordered from Eurofins. The probes were labeled 5′ and 3′ with digoxigenin (DIG). Following sequences were used for the sense and antisense strand probes:

5′-[DIG]-GAATGCTGCTGCTGCTGCTGCTGCTGCTG CTG-[DIG]-3′
5′-[DIG]-CAGCAGCAGCAGCAGCAGCAGCAGCAGC ATTC-[DIG]-3′.

### Small-Pool PCR

Amplification of the repeat containing DNA was modified after the original protocol by Monckton et al. (1995a). Prior to the polymerase chain reaction (PCR) DNA was diluted to a final amount of 50–300 pg per reaction. It is important to note that we did use a polymerase without proofreading activity for the small pool PCR, as the usage of proof-reading polymerases resulted in insufficient amplification of the repeat containing alleles. PCR reactions were performed in a total volume of 50 μl per reaction. The reaction mix contained 1.25 U AmpliTaq^®^ polymerase (AppliedBiosystems), appropriately diluted reaction buffer, 10 mM dNTPs, 100 μM of each primer (DM-A and DM-BR; Monckton et al., 1995a), and ddH_2_O. Primer sequences used are following:

Forward primer: 5′-CAGTTCACAACCGCTCCGAGC-3′
Reverse primer: 5′-CGTGGAGGATGGAACACGGAC-3′

Following settings were used for the PCR:

$$\begin{array}{l} \begin{array}{ccc} \text{Initial denaturation}: & {\text{95}}^{{}^{\circ}}\text{C} & \text{240s} \end{array}\\ \left(\begin{array}{lll} \text{Denaturation}: & {\text{95}}^{{}^{\circ}}\text{C} & \text{45 s}\\ \text{Annealing}: & {\text{68}}^{{}^{\circ}}\text{C} & \text{45 s}\\ \text{Extension}: & {\text{72}}^{{}^{\circ}}\text{C} & \text{180 }{\text{s}}^{\text{*}} \end{array}\right)\begin{array}{l} \text{28 cycles} \end{array}\\ \begin{array}{ccc} \text{Final extension}: & {\text{72}}^{{}^{\circ}}\text{C} & \text{600 s} \end{array} \end{array}$$

^∗^Time increment of 15 s per cycle.

The PCR was performed in a Biometra Tadvanced thermocycler (Analytik Jena).

### Gel Electrophoresis

The PCR products were size-separated by gel electrophoresis. For this a 1% agarose gel of 14 cm length was prepared using Tris-acetate-EDTA (TAE) buffer. The gel was loaded with 30 μl of PCR product mixed with 10 μl of loading buffer. Initial the electrophoresis was run for 10 min at 100 V (volt), followed by 60 min at 140 V.

### Southern Blot

After the electrophoresis the DNA was transferred via vacuum blot to a nylon membrane (Amersham Hybond^TM^-XL). The gel was washed in ddH_2_O and placed in depurination buffer for 15 min. After the depurination and a washing step with ddH2O the gel was placed for 30 min in denaturation buffer. Before the gel was equilibrated in 20× SSC buffer for 10 min it was incubated two times in neutralizing buffer for 15 min. The equilibrated membrane (in 20× SSC) was placed on the blotting apparatus (vacuum blot, Analytik Jena) and on top the gel. Stepwise the low vacuum of 90–100 mBar was established. During the blotting phase of 1–1.5 h 20× SSC buffer was constantly added to the top of the gel. Following this, the membrane was equilibrated for 2 min in 2× SSC buffer and then dried for 5 min at 65°C (UVP Hybrilinker Oven, Analytik Jena). Following the drying process, the DNA was cross-linked via UV-light (UVP Hybrilinker Oven, Analytik Jena). Solutions were prepared the following:

• Depurination buffer HCl 0.25 M
• Denaturation buffer NaCl 1.5 M; NaOH 0.5 M
• SSC buffer (20×) NaCl 3 M; Ma-Citrate 0.3 M (pH 7.5)
• Neutralizing buffer NaCl 1.5 M; Tris/HCl 0.5 M; EDTA 1 mM (pH 7.2).

### Hybridization and Detection

The membrane was transferred into a prewarmed hybridization tube with 10 ml PerfectHyb^TM^ Plus hybridization buffer (Sigma Aldrich) and equilibrated for 15–30 min. In the meantime, 20 μl of the two probes (9 ng/μl) where incubated with 80 μl ddH_2_O for 10 min at 100°C. After the denaturation step the probes where immediately placed on ice for 5 min and then transferred into the hybridization solution. After the overnight incubation the membrane was washed for 20 min at 65°C with prewarmed (65°C) washing solution (1× SSC + 0.2% SDS). After the second washing step the membrane was equilibrated for 5 min in wash-buffer (100 mM maleic acid + 150 mM NaCl + 0.3% Tween20 pH 7.5). Blocking solution (Roche DIG-detection Kit) was set up in maleic buffer (100 mM maleic acid + 150 mM NaCl pH 7.5) and then put on the membrane for 30 min. The DIG-antibody conjugate (Roche DIG-detection Kit) was diluted in blocking solution and the membrane was incubated for another 30 min. Following two 15 min washing steps the membrane was equilibrated in detection buffer (Roche DIG-detection Kit) for 5 min. The membrane was incubated for 15 min at 37°C in the detection solution (Roche DIG-detection Kit) before the signals were detected for 10 min using an Odyssey^®^ Fc imaging system (Licor).

### Calculation of the Repeat Length

To calculate the actual repeat length the image studio^TM^ software (Licor) was used to measure the size of the bands detected in samples based on the size of the marker bands. Based on these data the following formula was used:

$$\text{x}=\frac{\text{y}-\text{z}}{3}+\text{i}$$

x = number of repeats.

y = fragment length (measured using the image studio^TM^ software, Licor).

z = flanking gene sequence (311 bp).

i = number of the repeats within the marker sequence (20 repeats).

## Results

**FIGURE 2:**
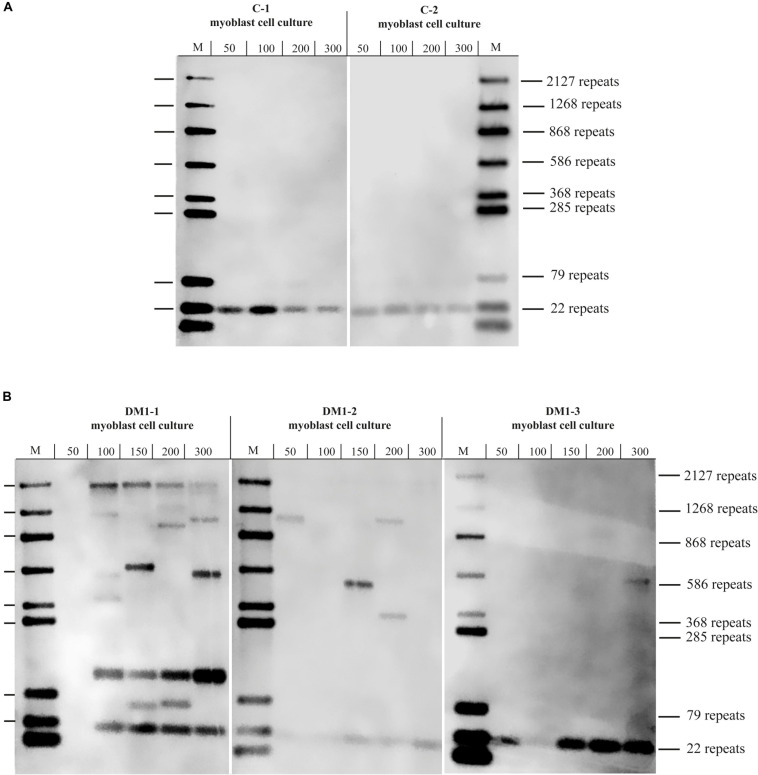
Southern blot for **(A)** control and **(B)** DM1 primary myoblast cultures showing the different with the amount of used DNA in pg (as input for the small pool PCR) shown. M = size-marker.

Using two control myoblast cultures we could detect small repeats with the calculated sizes of about 20 CTG-repeats for both (Figure 2A). Those repeats are within the range expected for normal alleles (up to 35 CTG-repeats). In the three DM1 myoblast cultures tested (Figure 2B), we could detect small CTG-repeats (about 20 CTG-repeats) in all of them. This band corresponds to the size of the wildtype allele. For the culture DM1-1 (left) we found several bands ranging from ∼50 to >2,000 CTG-repeats. The presence of this range of repeat length could indicate a mosaic situation in the muscle of this patient. In the culture DM1-2 (middle) we could also identify extended CTG-repeats in addition to the wildtype allele. Here the range is between 300 and 1,000 CTG-repeats. In DM1-3 (right) we only got one band for an extended allele of about 600 CTG-repeats.

**FIGURE 3:**
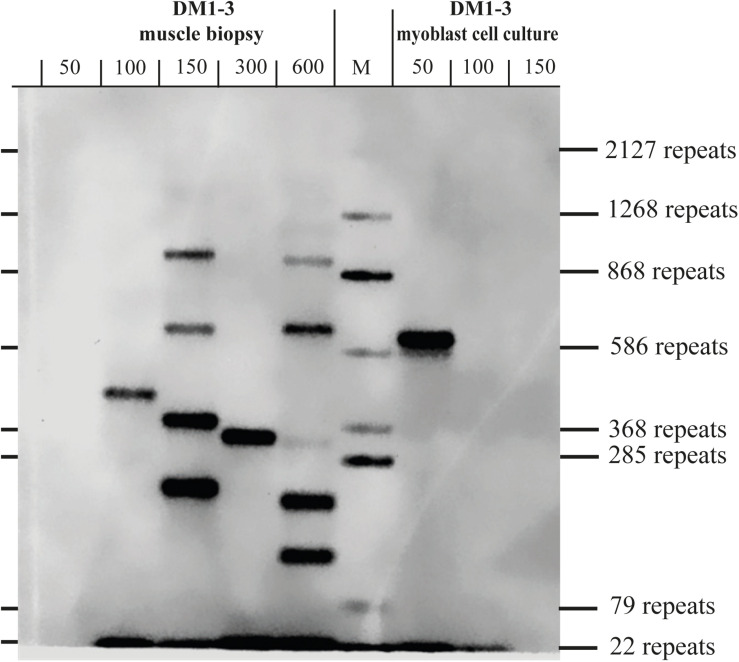
Southern blot comparing DNA derived from a muscle biopsy and a myoblast culture of the same DM1 patient (both samples taken at the same time). For each lane the amount of used DNA in pg (as input for the small pool PCR) is shown. M = size-marker.

To test if the repeat length in primary myoblast cultures is comparable to the muscle biopsy they were grown from, we tested material from the cryo-preserved biopsy of DM1-3 which was part of the biopsy the cell line was grown from and compared directly to the myoblast culture (Figure 3). For the myoblasts (right side of the blot) we got again a band showing about 600 repeats. For the muscle biopsy we got a range from ∼100 to ∼1,000 CTG-repeats for the mutant allele. The wildtype allele of about 20 CTG-repeats was detectable in both samples.

## Conclusion

With our modified method, we can successfully identify extended CTG-repeats in primary human myoblast cultures in comparison to DNA extracted from the original muscle specimen it was grown from. We show that there is a range of different repeat lengths in myoblast cultures. Thus, human myoblast cultures reflect rather well the repeat-length of mature muscle. Furthermore, it seems that the repeat length in myoblasts and muscle does, for the patient samples tested, not differ significantly from each other. Consequently, we show that primary human DM1 myoblast cultures are a well-suited model to investigate repeat-length related preclinical aspects of the DM1 pathology and are a useful platform to do first-line treatment interventions.

Presently, there are several distinct methods for the detection of CTG-repeats published (Warner et al., 1996; Kamsteeg et al., 2012; Singh et al., 2014; Turner and Hilton-Jones, 2014; Leferink et al., 2019). Most of them require sophisticated and often expensive equipment like capillary sequencers or a laboratory equipped for the usage of radioactive material. The modified method described here is an alternative method to detect CTG-repeat length, which is useful when only limited sample material and no special equipment is available. The usage of PCR-based DNA amplification is a limiting factor as the length of DNA, that can be synthesized by polymerases, is limited. However, this type of limitation also applies for any other method using polymerase-depending amplification.

## Data Availability Statement

The original contributions presented in the study are included in the article/supplementary material, further inquiries can be directed to the corresponding author/s.

## Ethics Statement

The studies involving human participants were reviewed and approved by the Ethical Review Committee at the Ludwig-Maximilians-University, Munich, Germany. The patients/participants provided their written informed consent to participate in this study.

## Author Contributions

SH, BS, and PM contributed to the conception and design of the experiments. PM and SH wrote the manuscript. SH, RM, and LK performed the experiments. All authors contributed to the article and approved the submitted version.

## Conflict of Interest

The authors declare that the research was conducted in the absence of any commercial or financial relationships that could be construed as a potential conflict of interest.

## References

[B1] Arandel L. Polay Espinoza M. Matloka M. Bazinet A. De Dea Diniz D. Naouar N. (2017). Immortalized human myotonic dystrophy muscle cell lines to assess therapeutic compounds. *Dis. Model. Mech.* 10 487–497. 28188264 10.1242/dmm.027367 PMC5399563

[B2] Bigot A. Klein A. F. Gasnier E. Jacquemin V. Ravassard P. Butler-Browne G. (2009). Large CTG repeats trigger p16-dependent premature senescence in myotonic dystrophy type 1 muscle precursor cells. *Am. J. Pathol.* 174 1435–1442. 10.2353/ajpath.2009.080560 19246640 PMC2671374

[B3] Brook J. D. McCurrach M. E. Harley H. G. Buckler A. J. Church D. Aburatani H. (1992). Molecular basis of myotonic dystrophy: expansion of a trinucleotide (CTG) repeat at the 3′ end of a transcript encoding a protein kinase family member. *Cell* 68 799–808. 10.1016/0092-8674(92)90154-5 1310900

[B4] Brunner H. G. Brüggenwirth H. T. Nillesen W. Jansen G. Hamel B. C. Hoppe R. L. (1993). Influence of sex of the transmitting parent as well as of parental allele size on the CTG expansion in myotonic dystrophy (DM). *Am. J. Hum. Genet.* 53 1016–1023. 8213829 PMC1682295

[B5] Cumming S. A. Jimenez-Moreno C. Okkersen K. Wenninger S. Daidj F. Hogarth F. (2019). Genetic determinants of disease severity in the myotonic dystrophy type 1 OPTIMISTIC cohort. *Neurology* 93 e995–e1009. 31395669 10.1212/WNL.0000000000008056 PMC6745735

[B6] De Antonio M. Dogan C. Hamroun D. Mati M. Zerrouki S. Eymard B. (2016). Unravelling the myotonic dystrophy type 1 clinical spectrum: a systematic registry-based study with implications for disease classification. *Rev. Neurol.* 172 572–580. 10.1016/j.neurol.2016.08.003 27665240

[B7] Echenne B. Bassez G. (2013). Congenital and infantile myotonic dystrophy. *Handb. Clin. Neurol.* 113 1387–1393. 10.1016/b978-0-444-59565-2.00009-5 23622362

[B8] Faustino N. A. Cooper T. A. (2003). Pre-mRNA splicing and human disease. *Genes Dev.* 17 419–437. 10.1101/gad.1048803 12600935

[B9] Gomes-Pereira M. Bidichandani S. I. Monckton D. G. (2004). Analysis of unstable triplet repeats using small-pool polymerase chain reaction. *Methods Mol. Biol.* 277 61–76. 15201449 10.1385/1-59259-804-8:061

[B10] Hayflick L. (1965). The limited in vitro lifetime of human diploid cell strains. *Exp. Cell Res.* 37 614–636. 10.1016/0014-4827(65)90211-9 14315085

[B11] Hintze S. Knaier L. Limmer S. Schoser B. Meinke P. (2018). Nuclear envelope transmembrane proteins in myotonic dystrophy type 1. *Front. Physiol.* 9:1532. 10.3389/fphys.2018.01532 30425655 PMC6218431

[B12] Ho G. Carey K. A. Cardamone M. Farrar M. A. (2019). Myotonic dystrophy type 1: clinical manifestations in children and adolescents. *Arch. Dis. Child.* 104 48–52. 10.1136/archdischild-2018-314837 29871899

[B13] Kamsteeg E. J. Kress W. Catalli C. Hertz J. M. Witsch-Baumgartner M. Buckley M. F. (2012). Best practice guidelines and recommendations on the molecular diagnosis of myotonic dystrophy types 1 and 2. *Eur. J. Hum. Genet.* 20 1203–1208. 10.1038/ejhg.2012.108 22643181 PMC3499739

[B14] Leferink M. Wong D. P. W. Cai S. Yeo M. Ho J. Lian M. (2019). Robust and accurate detection and sizing of repeats within the DMPK gene using a novel TP-PCR test. *Sci. Rep.* 9:8280. 10.1038/s41598-019-44588-3 PMC6547747 31164682

[B15] LoRusso S. Weiner B. Arnold W. D. (2018). Myotonic dystrophies: targeting therapies for multisystem disease. *Neurotherapeutics* 15 872–884. 10.1007/s13311-018-00679-z 30341596 PMC6277298

[B16] Meinke P. Hintze S. Limmer S. Schoser B. (2018). Myotonic dystrophy-a progeroid disease? *Front. Neurol.* 9:601. 10.3389/fneur.2018.00601 30140252 PMC6095001

[B17] Meola G. Cardani R. (2015). Myotonic dystrophies: an update on clinical aspects, genetic, pathology, and molecular pathomechanisms. *Biochim. Biophys. Acta* 1852 594–606. 10.1016/j.bbadis.2014.05.019 24882752

[B18] Monckton D. G. Wong L. J. Ashizawa T. Caskey C. T. (1995a). Somatic mosaicism, germline expansions, germline reversions and intergenerational reductions in myotonic dystrophy males: small pool PCR analyses. *Hum. Mol. Genet.* 4 1–8. 10.1093/hmg/4.1.1 7711720

[B19] Nakamori M. Sobczak K. Puwanant A. Welle S. Eichinger K. Pandya S. (2013). Splicing biomarkers of disease severity in myotonic dystrophy. *Ann. Neurol.* 74 862–872. 10.1002/ana.23992 23929620 PMC4099006

[B20] Overend G. Légaré C. Mathieu J. Bouchard L. Gagnon C. Monckton D. G. (2019). Allele length of the DMPK CTG repeat is a predictor of progressive myotonic dystrophy type 1 phenotypes. *Hum. Mol. Genet.* 28 2245–2254. 10.1093/hmg/ddz055 31220271 PMC6586140

[B21] Pantic B. Borgia D. Giunco S. Malena A. Kiyono T. Salvatori S. (2016). Reliable and versatile immortal muscle cell models from healthy and myotonic dystrophy type 1 primary human myoblasts. *Exp. Cell Res.* 342 39–51. 10.1016/j.yexcr.2016.02.013 26905645

[B22] Plantié E. Migocka-Patrzałek M. Daczewska M. Jagla K. (2015). Model organisms in the fight against muscular dystrophy: lessons from drosophila and Zebrafish. *Molecules* 20 6237–6253. 10.3390/molecules20046237 25859781 PMC6272363

[B23] Redman J. B. Fenwick R. G. Jr. Fu Y. H. Pizzuti A. Caskey C. T. (1993). Relationship between parental trinucleotide GCT repeat length and severity of myotonic dystrophy in offspring. *Jama* 269 1960–1965. 10.1001/jama.1993.03500150072029 8464127

[B24] Renault V. Piron-Hamelin G. Forestier C. DiDonna S. Decary S. Hentati F. (2000). Skeletal muscle regeneration and the mitotic clock. *Exp. Gerontol.* 35 711–719. 10.1016/s0531-5565(00)00151-0 11053661

[B25] Renna L. V. Bosè F. Iachettini S. Fossati B. Saraceno L. Milani V. (2017). Receptor and post-receptor abnormalities contribute to insulin resistance in myotonic dystrophy type 1 and type 2 skeletal muscle. *PLoS One* 12:e0184987. 10.1371/journal.pone.0184987 28915272 PMC5600405

[B26] Savkur R. S. Philips A. V. Cooper T. A. (2001). Aberrant regulation of insulin receptor alternative splicing is associated with insulin resistance in myotonic dystrophy. *Nat. Genet.* 29 40–47. 10.1038/ng704 11528389

[B27] Sicot G. Gomes-Pereira M. (2013). RNA toxicity in human disease and animal models: from the uncovering of a new mechanism to the development of promising therapies. *Biochim. Biophys. Acta* 1832 1390–1409. 10.1016/j.bbadis.2013.03.002 23500957

[B28] Singh S. Zhang A. Dlouhy S. Bai S. (2014). Detection of large expansions in myotonic dystrophy type 1 using triplet primed PCR. *Front. Genet.* 5:94. 10.3389/fgene.2014.00094 24795756 PMC4006065

[B29] Souidi A. Zmojdzian M. Jagla K. (2018). Dissecting pathogenetic mechanisms and therapeutic strategies in drosophila models of myotonic dystrophy type 1. *Int. J. Mol. Sci.* 19:4104. 10.3390/ijms19124104 30567354 PMC6321436

[B30] Thornton C. A. Johnson K. Moxley R. T. III (1994). Myotonic dystrophy patients have larger CTG expansions in skeletal muscle than in leukocytes. *Ann. Neurol.* 35 104–107. 10.1002/ana.410350116 8285579

[B31] Turner C. Hilton-Jones D. (2014). Myotonic dystrophy: diagnosis, management and new therapies. *Curr. Opin. Neurol.* 27 599–606. 10.1097/wco.0000000000000128 25121518

[B32] Udd B. Krahe R. (2012). The myotonic dystrophies: molecular, clinical, and therapeutic challenges. *Lancet Neurol.* 11 891–905. 10.1016/s1474-4422(12)70204-1 22995693

[B33] Warner J. P. Barron L. H. Goudie D. Kelly K. Dow D. Fitzpatrick D. R. (1996). A general method for the detection of large CAG repeat expansions by fluorescent PCR. *J. Med. Genet.* 33 1022–1026. 10.1136/jmg.33.12.1022 9004136 PMC1050815

[B34] Wenninger S. Montagnese F. Schoser B. (2018). Core clinical phenotypes in myotonic dystrophies. *Front. Neurol.* 9:303. 10.3389/fneur.2018.00303 29770119 PMC5941986

[B35] Wheeler T. M. (2008). Myotonic dystrophy: therapeutic strategies for the future. *Neurotherapeutics* 5 592–600. 10.1016/j.nurt.2008.08.001 19019311 PMC4514697

[B36] Wong L. J. Ashizawa T. Monckton D. G. Caskey C. T. Richards C. S. (1995b). Somatic heterogeneity of the CTG repeat in myotonic dystrophy is age and size dependent. *Am. J. Hum. Genet.* 56 114–122. 7825566 PMC1801291

